# Determinants of the quality of care relationships in long-term care - a participatory study

**DOI:** 10.1186/s12913-019-4195-x

**Published:** 2019-06-14

**Authors:** Aukelien Scheffelaar, Michelle Hendriks, Nanne Bos, Katrien Luijkx, Sandra van Dulmen

**Affiliations:** 1 Nivel (Netherlands institute for health services research), PO Box 1568, 3500 BN Utrecht, The Netherlands; 2 Radboud Institute for Health Sciences, Department of Primary and Community Care, Radboud university medical center, Nijmegen, The Netherlands; 3 Reinier van Arkel, Den Bosch, The Netherlands; 4 Tilburg School of Social and Behavioral Sciences, Tranzo, Tilburg University, Tilburg, The Netherlands; 5 Faculty of Health and Social Sciences, University of South-Eastern Norway, Drammen, Norway

**Keywords:** Care relationship, Client-professional relationship, Long-term care, Quality of care, Client perspective, Professional perspective, Qualitative research, Client participation

## Abstract

**Background:**

The quality of the care relationship between a client and a professional is important in long-term care, as most clients depend on support for a lengthy period. The three largest client groups who receive long-term care in the Netherlands are older adults who are physically or mentally frail, people with mental health problems and people with intellectual disabilities. There is little clarity about how generic and variable the determinants of the quality of care relationships are across these client groups. The aim of this study is to explore and compare the determinants of the quality of care relationships in these three client groups in long-term care.

**Methods:**

This participatory study involving clients as co-researchers was held in three healthcare organizations, each providing long-term care to one client group. The research was conducted by three teams consisting of researchers and co-researchers. We interviewed clients individually and professionals in focus groups. The focus was on care relationships with professionals where there is weekly recurring contact for at least 3 months. Clients and professionals were selected using a convenience sample. The interviews were coded in open, axial and selective coding. The outcomes were compared between the client groups.

**Results:**

The study sample consisted of 30 clients and 29 professionals. Determinants were categorized into four levels: client, professional, between client and professional, and context. The findings show that the majority of the determinants apply to the care relationships within all three client groups. At the professional level, eleven generic determinants were found. Eight determinants emerged at the client level of which two were found in two client groups only. At the level between a client and a professional, six determinants were found of which one applied to mental healthcare and disability care only. Five determinants were found at the contextual level of which two were specific for two client groups.

**Conclusions:**

The study yielded a variety of determinants that came to the fore in all three client groups in long-term care. This suggests that including a homogenous client group from a single care setting is not necessary when studying the quality of long-term care relationships.

**Electronic supplementary material:**

The online version of this article (10.1186/s12913-019-4195-x) contains supplementary material, which is available to authorized users.

## Background

The importance of the quality of care relationships between professionals and clients in long-term care, where clients depend on support for their basic needs in daily life for longer periods, is well documented [1–3]. The care relationship is seen as serving several purposes. A good quality of a care relationship correlates with greater life satisfaction for clients [4]. A care relationship can also provide opportunities for growth and development for a client as well as a professional, and can help clients in their progression towards independence [5, 6]. Moreover, a care relationship offers clients recognition, created by the awareness and acknowledgment of sharing a fundamental likeness as human beings [5].

Although the quality of the care relationship is clearly important for both clients and professionals, there is little clarity as yet about the nature of these relationships [2]. And with good reason: the variable and fluctuating nature of care relationships makes them hard to define and study [5]. Some authors have defined care relationships within current theories such as care ethics [7], relational ethics [8] or relationship-centred care [9]. Other authors have addressed a single aspect of the care relationship, such as professional friendship [3], therapeutic relationship [10, 11], autonomy [12], client engagement [13], power dynamics [13] or communicative barriers [14]. Given these multiple components of a relationship, a care relationship should be seen as a multidimensional construct [10, 15].

Research findings show that the quality of care relationships is not yet optimal for all those involved. Multiple reasons are reported for the low quality of care relationships; stigmatization [6, 7], discontinued relationships with professionals who are leaving, professionals lacking time, and the negative impact of heavy workloads [6, 10, 16–20]. Clients also mentioned untrustworthy professionals, and staff who rejected clients’ opinions and discouraged them, resulting in clients feeling dismissed and ignored [6]. In a study by Eriksen (2013), clients with mental health problems reported feeling detachment in care relationships, experiencing a lack of interest, unwillingness to be understood and indifference or even hostility from professionals [21]. Moreover, discrimination and language barriers diminished the quality of care relationships between care workers and older, physically or mentally frail people [22].

Determining the quality of care relationships from clients’ perspectives gives clients and professionals insights into areas for improvement. This can help professionals improve their performance and aim to achieve high-quality care relationships [2, 18, 23]. We recently carried out a systematic review to map out the determinants of the quality of care relationships between clients and professionals in long-term care [24]. This review focused on three client groups: physically or mentally frail older adults, people with mental health problems and people with intellectual disabilities. The review suggested a substantial number of determinants that may apply to more than one client group. Until now, all studies have, however, focused on one single client group in long-term care. As the majority of determinants may be expected to be generic and not client-group specific, such a focus on specific client groups might not necessarily be needed for identifying gaps in the quality of care relationships in long-term care [24].

There is, however, no instrument available for monitoring and evaluating the quality of care relationships that can be used within various client groups in long-term care. Such a generic instrument can make it easier for care organizations to learn from good practices in other care organizations and to improve the exchange of knowledge. Insight is needed into the generic determinants of the quality of care relationships before such a generic method can be compiled.

The aim of this study is therefore to explore the determinants of the quality of the care relationship between a client and a professional in long-term care and how generic these determinants are among different client groups. The central question of this article is: What are determinants of the quality of a care relationship in long-term care according to clients and care professionals? The client groups included are physically or mentally frail older adults, people with mental health problems and people with intellectual disabilities, i.e. the largest client groups in the Netherlands receiving long-term care. We looked at the perspectives of both clients and professionals, as clients and professionals have been found to use different definitions and have different perspectives on care relationships [2].

Clients have unique experiential knowledge derived from personal experiences and their perceptions on receiving care [25, 26]. Therefore, the clients’ perspective was included in this research by performing participatory research. In participatory research, clients are invited to join the research team as co-researchers and contribute by participating in several stages of a research project [27–29].

## Methods

### Study design and setting

The participatory study took place in three organizations providing long-term care to three distinct client groups. One care organization provided care to physically or mentally frail older adults, the second organization provided care to people with mental health problems and the third provided care to people with intellectual disabilities. All three care organizations provided care in both inpatient and outpatient care settings. The three care organisations serve a large client population with a diversity of recurring care needs. Their client population entails more than 2000 clients, and more than 2000 care professionals are employed at each care organisation. The three large care organisations were selected to increase the transferability of the findings to other care organisations, as these organisations were largely representative for the client populations of long-term care in the Netherlands. The study employed an exploratory qualitative design in order to make the contributions of co-researchers meaningful and to let them use terminology according to their preferences. The data collection method involved individual semi-structured interviews with clients and semi-structured focus groups with professionals in each care organization. A protocol paper for the overall study has been published elsewhere [30].

### Participatory research method

Participatory research is known to improve the quality, the relevance and usefulness of the findings, for democratizing research and for empowering the clients who participate [25, 31]. The quality of the research is expected to improve because co-researchers are involved in the formulation of the questions, the data collection and the analysis of the data which enhances the (content) validity and relevance of the results. For example, when co-researchers formulate their questions in interviews on care relationships, they are likely to use plain language and use questions that resonate a client perspective. The contributions of co-researchers are likely to improve the understanding of clients, and make clients feel more comfortable during the interviews.

A research team was composed in each care organization consisting of three to four co-researchers and two researchers. Co-researchers had care experience with the specific care organization in which the research took place. Co-researchers followed a training course consisting of three meetings about qualitative research, practicing semi-structured interviewing and making agreements about confidentiality and support. The topics covered by the training were tailored to the needs and wishes of the co-researchers.

**Fig. 1:**
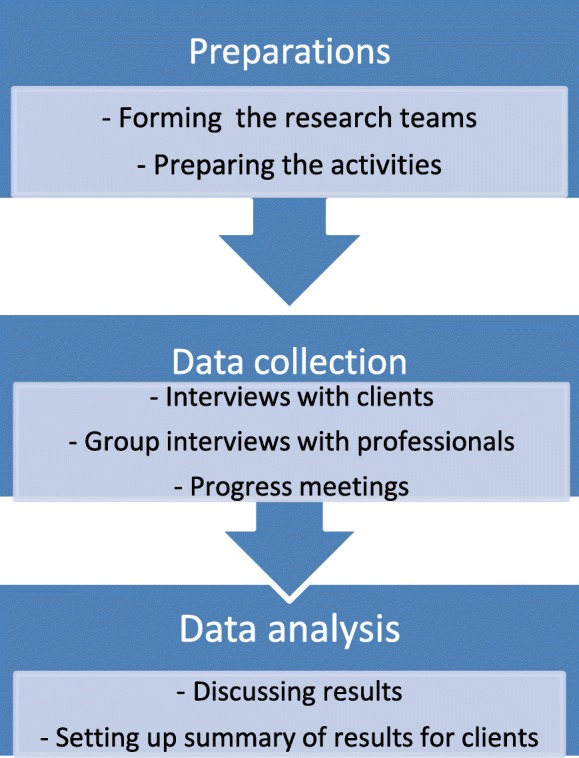
Design of the research teams activities

The three research teams were involved from the beginning of the study until the end. First, we created a topic list with the research team and carried out preparatory activities such as setting up the invitation for respondents. Co-researchers helped recruit the participants. Each interview was conducted by one co-researcher and one researcher and the division of roles was decided upon before each interview took place. The research team gathered at work meetings during which we talked about the initial experiences with interviewing and cooperation. In later work meetings, we spoke about the interview results. We also discussed the summary of findings we sent to the respondents (Fig. 1).

### Recruitment of respondents

The study focused on relationships between clients and professionals having weekly recurring contact in long-term care for at least 3 months. Clients received care in their own home (outpatient) or within the care organization in which they reside (inpatient). Most clients received care at least once every week, but the assistance for some outpatient clients with long-term mental healthcare was more loosely planned. The study focused on care relationships with professionals who see clients most often to provide assistance, supporting care and physical care, e.g. care aides, personal carers and various types of nurses.

Clients and professionals were selected using a convenience sample. Clients who met our inclusion criteria were selected by the research team in consultation with client councils and the contact person of each care organization and invited to take part by the research team. There was aimed for variation in terms of client characteristics such as type and intensity of care, sex, age, and whether the care was intramural or extramural. All clients were 18 years or older (no upper limit), and able to communicate verbally in Dutch. Clients received a verbal and written invitation for an interview. In some instances clients were informed by their personal professional at first and asked if they could be contacted before the research team invited them. This was only done when the professionals preferred to inform the client themselves first. In some other instances the legal representatives of people with intellectual disabilities were asked for permission first.

Variation between professionals was aimed at as well, in variables such as years of work experience, sex, working in an inpatient or outpatient setting, and type of function (e.g. care aides, personal assistants, different types of nurses and activity supervisors). Professionals providing more remote physical and supporting care were not included, such as clinicians, psychiatrists and general practitioners. Caregivers who provide informal care were also not included. The professionals were selected and invited by the researcher (AS) in close cooperation with the contact person for each care organization. Professionals received an invitation for a focus group by e-mail.

### Individual interviews with clients

Clients were interviewed face-to-face by a pair consisting of one co-researcher and one researcher (AS, NB or field researcher) in the residence of a client or a meeting room of the care organization, depending on the preference of the client. The interviews involved questions about clients’ experiences with their care relationship with one or more professionals. The interviewers followed up on the broader opening questions by asking clients additional questions in order to probe more deeply on the experiences a client tells about and to provide as much detail as possible. In this way, a client was able to determine what topics related to the care relationship he or she told about. The topic list is included in Additional file 1. Depending on the concentration span of each client, the interview lasted between 30 and 60 min. Data collection of the interviews ended when saturation was reached and no new themes emerged from the data.

### Focus groups with professionals

In every care organization, one or two focus groups were held with professionals. The focus groups took about 2 h. One researcher (AS) and one or two co-researchers had the role of moderator. First, a poster assignment was done in pairs of two professionals. Professionals discussed and indicated what affects a care relationship, either positively or negatively. Professionals also wrote down opportunities they saw for improving care relationships and other ideas they had. This was followed by a group discussion based on the ideas and experiences the professionals had noted in pairs. The moderators asked probing questions and ensured that all professionals had the opportunity to speak. They also asked other respondents about their experiences and reactions when a determinant was mentioned by one care professional. All interviews and focus groups were audio recorded with the permission of the respondents.

### Definition of “determinant”

This study is focused on determinants of the quality of a care relationship, by exploring what might influence the quality of care relationships from the viewpoints of clients and professionals. A determinant is therefore defined as an aspect related to the quality of a care relationship which might determine a care relationship in a positive or negative manner. Since every care relationship is developed and maintained in its own unique way, a determinant might have a variable and context specific elaboration on different care relationships. Two determinants might be also conflicting or incompatible to one another.

### Data analysis

The focus groups and interviews were transcribed verbatim and analysed in three phases: open coding, axial coding and selective coding [32]. The data analysis method was inspired by Interpretative Phenomenological Analysis, which is centred on the respondents’ experiences and the meaning they assign to those experiences [33]. The analysis process was carried out for each care organization separately at first.

#### Open coding

Each research team discussed research findings in a work meeting. Co-researchers shared their own experiences and interpretations in the research team and the most important themes they heard in interviews. The topics discussed by the research team were written on a flip chart that was the start of the open coding process. Two researchers (AS and MH) then read the interviews collected so far and identified themes that emerged from the interviews. They used the themes identified in the work meetings as inductive codes. In a meeting, the two researchers (AS and MH) composed a list of open codes and reached consensus on the formulation of themes. AS is employed as a PhD student and has a background in interdisciplinary social sciences. MH is educated in psychology and has 18 years of work experience in the field of health services research. Her research mainly focuses on patient’s experiences with health care.

#### Axial coding

Two researchers (AS and MH) independently analysed three interviews of each client group in MAXQDA. They worked from the codes to the data and determined whether the codes adequately covered the collected data. They created new codes when new themes emerged. In a meeting, the researchers (AS and MH) discussed differences in interpretations. In addition, they categorized interrelationships between themes that distinguished master codes from subcodes and adjusted the code tree.

#### Selective coding

The same two researchers analysed and discussed two interviews of each client group in MAXQDA independently using the adjusted code tree. When they disagreed on the interpretation of a fragment, they tried to reach consensus by discussion. In a meeting, the researchers adapted the code tree further and compared the three code trees of each sub-study. Formulations and interrelationships between codes were made uniform where possible. The main findings were discussed with two other researchers involved in performing the interviews (NB and a field researcher), paying specific attention to differences and similarities between the three client groups in long-term care and the interrelationships between the determinants. All authors were informed about and involved in the broad picture obtained from the analysis during two discussion meetings. Finally, the interviews were checked again with the final code trees by AS.

### Ethical considerations

The study was submitted to the Medical Ethics Committee of the Radboud university medical center to decide whether the study needed formal approval. Given the Dutch Medical Research Involving Human Subjects Act, the Ethics Committee decided that extensive formal approval was not needed for this study.

All participants were given both written and verbal information about the study, including the purpose and procedures, confidentiality of individual interviews or focus groups, the voluntary nature of participation and the opportunity to withdraw at any time. Interested clients and professionals participated after completing a consent form. In the interviews with clients, we also adopted a ‘process consent’ approach, meaning that we constantly observed whether consent was still present by paying attention to verbal and nonverbal indications of reluctance or hesitation to participate [35].

To ensure a meaningful participation of co-researchers, support was provided in several ways and basic agreements for cooperation and confidentiality were drawn up by the research team.

## Results

The sample for the study consisted of a total of 30 clients and 29 professionals. The clients interviewed varied with respect to the intensity of care, client group, care setting (inpatient or outpatient) and background characteristics such as age and sex. Professionals were employed in various professions and varied in sex. See Table 1 for an overview of the respondents per client group.

**Table 1:** Respondents of the study

Type of care	Clients	Professionals
Mental healthcare	11	11
Intellectual disability care	9	8
Elderly care	10	10
Sex (female / male)	13 / 17	21 / 8
Care setting (inpatient / outpatient)	16 / 14	16 / 8 * 5 professionals worked for an inpatient and as well as an outpatient setting in disability care.

Based on the qualitative data, the determinants could be broken down into four levels: client, professional, between a client and professional, and contextual. Each level makes clear which actor is influencing these determinants of the quality of care relationships to the utmost extent. The data analysis showed that most determinants came to the fore in all three client groups. We will therefore discuss the generic determinants first, followed by the determinants that emerged in one or two client groups only. We define a determinant as generic when a determinant was mentioned in all client groups, by clients and/or professionals. The determinants are not all generic in the sense that both clients and professionals of each client group discussed a determinant. Only a few determinants were mentioned solely by either clients or professionals; most of these were at the contextual level. An overview of determinants is given in Table 2.

**Table 2:**
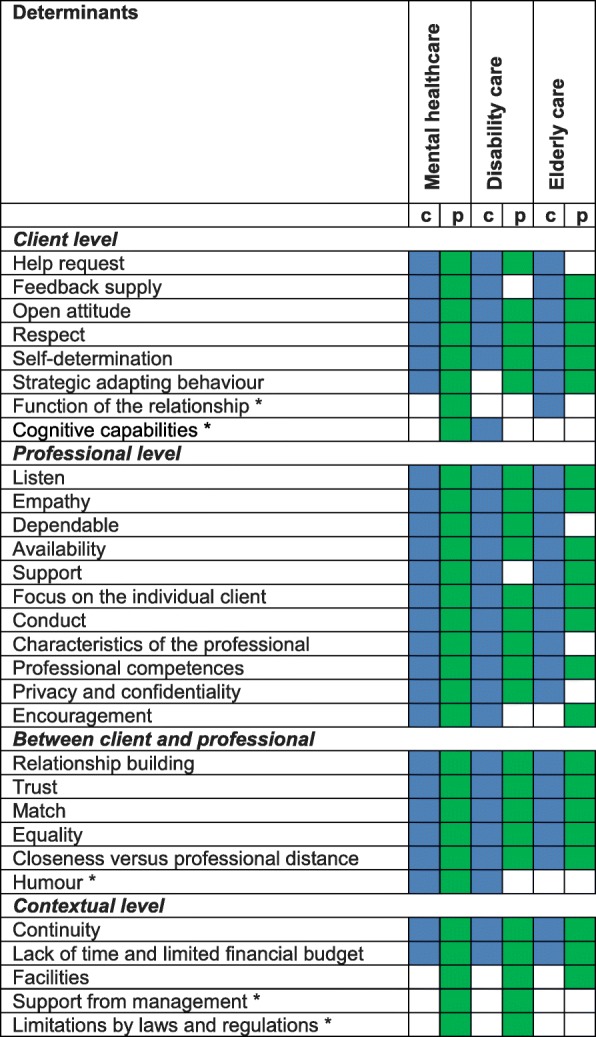
Determinants of the quality of a care relationship

*c* determinant mentioned by clients, *p* determinant mentioned by professionals

An asterisk (*) has been added to determinants that appeared to be specific for one or two client groups only

### Generic determinants

#### Client level

At the client level, six generic determinants were described by clients and/or professionals: help request, feedback supply, open attitude, respect, self-determination and strategic adapting behaviour.

- *Help request*

Clients and professionals suggested it is important that clients ask for help when they need it, in order to get appropriate support and care. Professionals explained that it is easier for them to provide the required support when clients tell them what they want. However, some clients found it difficult to ask a professional for assistance. Client 4, with an intellectual disability: ‘*“That’s what he [professional] always says too: you must come to me if there are any problems. Well, I find that difficult sometimes.”’*

- *Feedback supply*

Several clients and professionals mentioned that it is important that clients tell professionals what they appreciate and what can be improved in a care relationship. Some clients said that they share suggestions and express disapproval; others explicitly stated that they do not. One professional saw a tendency for older clients to increasingly provide feedback to professionals in comparison to some years earlier. Some other professionals explicitly ask for feedback to make it easier for clients to share their suggestions.

- *Open attitude*

Clients and professionals felt that an accessible and open attitude from the clients is very important as well. Client 30, receiving mental healthcare: *“How open you yourself are matters too, of course. Like, if you think ‘Uh-oh, here’s another new one’ and ‘Ooh, that’s scary’ then it’s going to be a barrier.”* According to the professionals, care relationships are negatively influenced by clients who distance themselves and who try to avoid contact. Less open attitudes on the part of clients could be related to negative experiences with care in the past, lack of language proficiency, emotional state, or coercive measures.

- *Respect*

Clients and professionals stated that a respectful approach from clients to professionals influences the quality of a care relationship positively. Clients referred to respect using phrases such as ‘decency’, ‘no insulting or name-calling’, ‘being polite’ and ‘taking the opinion and suggestions of a professional seriously’. One professional spoke about a client who acted unhelpfully and showed no respect at all, whom she asked to communicate in a more friendly way. A professional providing care for the elderly: *“You get the occasional client who absolutely snaps at you [...] and then I tell them that you can say whatever you want, but you can say it nicely. And he accepted that.”*

- *Self-determination*

Self-determination is about the control that a client has in a care relationship and the provision of care. Clients described the importance of having control over social aspects of life such as freedom to leave the care institution by themselves. Other clients had to get used to the fact that they had to play an active role in treatment or care, whereas they had expected that the professional would solve their problems for them.

- *Strategic adapting behaviour*

Clients mentioned they are trying to adapt their communication and wishes to the professionals and their capabilities at that moment. Client 16, receiving elderly care: *“You read the body language too, of course. You know, she’s feeling good today, or he’s had a tough time, or that one’s been busy. And if you respond to that a bit, it does make the communication a little easier.”* The majority of elderly clients said that they desire to be perceived as untroublesome and uncomplicated by professionals. Client 17, receiving elderly care: *“Well, I’ve not really got much to complain about. I don’t think I’m much trouble.”* Professionals of all client groups described clients varying from easy-going to some who are overly demanding.

### Professional level

Eleven generic determinants came to the fore at the professional level: listening, empathy, dependable, availability, support, focus on the individual client, conduct, characteristics of the professional, professional competences, privacy and confidentiality, and encouragement.

#### Listen

Clients and professionals stated that listening is an essential ability for professionals. Clients found it easier to tell a listening professional what bothers them, how they are feeling, and how their day was. Client 6, who has an intellectual disability: *“It’s nice to have someone I can get things off my chest with – what happened at work, or what annoyed me.”* According to clients, a listening care professional was better able to understand a client. By remembering important events or people in the life of a client, professionals can demonstrate that they have been listening.

#### Empathy

Some professionals immediately sense the mood of a client. Clients appreciated these watchful professionals. Client 23, receiving mental healthcare*: “She comes in and she can tell from my face how I’m feeling.”* Empathic professionals sense the state of a client very well and can also respond appropriately. Professionals said that they use their intuition to sense the mood of a client, and ask questions to verify the current mood or feelings of a client. A professional providing mental healthcare: *“Then you confirm it. ‘I reckon I can see or sense this – is that right?’”*

- *Dependable*

Being dependable is another essential characteristic of professionals. Dependable professionals fulfil their promises and meet the agreements that are made. Some clients described a trustworthy professional as someone who communicates completed decisions and progress made, transparently. Moreover, a dependable professional is truthful and does not deny what has been said or done. Client 6, intellectually disabled: *“If a professional makes a mistake, or denies something and says that she didn’t say it.”* A professional providing mental healthcare used the term ‘fairness’ in her description, and described dependable as *“Do what you say, and say what you’re doing.”*

- *Availability*

Availability comprises three related aspects: accessibility, quick responses and taking time for a client. Professionals said that they try to respond quickly when something happens or when a client calls for help. Accessibility is about clients feeling that they can reach a professional when they need help. Some clients said they felt they can go to a professional whenever needed. Many clients stated it was important that they felt a professional took time for them. This is something different from *having* time: a professional can take time for the care for a client while they are actually behind schedule.

- *Support*

Clients and professionals stated that support from professionals influences the quality of the relationship as well. Client 13, receiving elderly care: *“Those people [the professionals] give comfort, encouragement and support. That’s very important.”* Support can be expressed in different ways, like being the clients’ adviser, accompanying a client to a stressful appointment, giving useful advice or helping to find a solution for a client’s problem.

- *Focus on the individual client*

Clients and professionals underlined the importance of an individualised approach. It means that a client’s wishes and needs are taken into account. It also means that the timing and speed of care is adapted to a client. Instances described by professionals were time preferences, preferences for physical care and group activities, individually tailored conversations or in short: going the extra mile. A professional providing mental healthcare: *“That you can play it by ear, respond by doing whatever might help the patient at that moment.”* According to clients, showing interest for a client is key, chatting about the experiences, hobbies or the private situation of a client. Professionals reported various examples in which they paid personal attention: by greeting a client, small talk, or putting a hand on a clients’ shoulder to comfort them in their grief.

- *Conduct*

The way clients are treated was felt to be positive when the professional took a client seriously, had no prejudices towards the client, the manner and tone in which things were expressed felt right, or when a professional was kind and spontaneous towards a client. Professionals said that positive treatment includes transparency, authenticity, respect and a hospitable, cheerful and spontaneous attitude. Clients characterised negative attitudes as ignoring their wishes, detached, being rude or providing care in a domineering and unequal manner. Professionals referred to prejudices, arrogance and using unnecessary force. A professional providing disability care: *“Assessment, judging and pre-judging are all about not being open to the other person’s views and not picking up on them. That also applies to the ‘I know better’ attitude, because I’m your professional. And, well, then it all goes pear-shaped.”*

- *Characteristics of the professional*

Several characteristics of professionals were mentioned by clients, such as sex, age, years of work experience, and having similar features to the client or a relative. Professionals mentioned features such as self-reflection, job satisfaction, work experience and age difference. Some clients had a specific preference for one or a combination of individual characteristics, for example for a professional of the same age or the same sex. Client 3, intellectually disabled: *“Then you’re on the same wavelength.”* Other clients described having no such preferences.

- *Professional competences*

Clients valued proper, careful and high-quality care, support and assistance. Professionals underlined the importance of communication skills and knowing their own limits, as well as knowledge and skills specific to the client group. Clients with intellectual disabilities also referred to clear communication skills of professionals, including explanations and understandable words.

- *Privacy and confidentiality*

According to the clients, confidentiality and privacy included keeping client information confidential from other clients, their relatives or other professionals. Client 16, receiving elderly care: *“I know that I can tell them things and it will remain between us.”* Clients said that information was sometimes not written in their personal file when they asked their professional not to. Professionals distinguished between clients who had a formal representative and those who did not. When clients did have a formal representative, they were obliged to provide information to the representative, while in the other instances they provided information to family according to clients’ wishes.

- *Encouragement*

Encouragement was mentioned by several clients and professionals. Professionals were sometimes supporting a client to think of possible solutions of an issue or asked critical questions to encourage them to think things through from different viewpoints. Client 24, in mental healthcare: “*Then you get X’s critical questions again pretty often... She sometimes holds up a mirror so that you can take a look at yourself.”*

### Between client and professional

Five generic determinants were mentioned at the level between a client and professional: relationship building, trust, match, equality and closeness versus professional distance.

- *Relationship building*

According to both clients and professionals, building a relationship with a professional takes time, as professionals gradually get to know the client and their wishes. Some clients related relationship building to the development of trust in a care professional. After a while, clients started to share more delicate issues when they got to know and trust a professional. Professionals underlined the importance of the first contact with a client, they want to be hospitable and look for clients’ needs directly. They also felt that relationship building requires regular contact and investments in a care relationship by organising informal activities and doing something extra for clients.

- *Trust*

Trust was described as important by a fair number of clients, as a prerequisite for sharing thoughts and experiences with a professional. This determinant is related to the trustworthiness described for the professional level. Professionals believed trust will arise when there is mutual respect, they (as professionals) are trustworthy and reliable for clients because they keep promises and stick to the agreements that are made, and they book successes jointly with the clients. Professionals also related the determinant ‘trust’ to continuity created by the fixed assignment of a small number of professionals to each client.

- *Match*

Some clients felt a match with a professional from the first moment. A match means a client feels they can tell anything to a professional, trust the professional, and feels calm when the professional is present. When clients did not feel there was a match, they often did not like the professional and did not have a feeling that they could trust them. Client 5, intellectually disabled: *“There was a click right away, and that doesn’t then go away. And, well, if I don’t get that with someone straight off, then it takes ages before I trust the professional enough to say things to them.”* Professionals suggested that a match is not necessary for the care relationship with a client, but can indeed encourage relationship building and continuation of a care relationship.

- *Equality*

For several clients and professionals, equal positioning is important. Client 29, receiving mental healthcare: *“She didn’t act like she was above me. She was standing next to me. Close by, if you like.”* The examples provided include giving clients space to say what they prefer instead of interrupting immediately, making decisions together, professionals giving suggestions instead of orders. Professionals tried to achieve an equal position by sharing personal experiences and making decisions together with a client. In mental healthcare, professionals believed coercive measures are counterproductive for equal positioning, because clients do not have the power to decide where to go and what they want anymore. There were some instances mentioned in which disagreement between a client and professional occurred, but was not seen as a problem due to the equal positioning. A professional providing mental healthcare: *“If someone disagrees [...] I don’t say that they should see it my way. I try to let them understand why I think like I do. You mostly then find a compromise or you can at least agree to disagree.”*

#### Closeness versus professional distance

Some clients mentioned establishing more informal relationships with a professional. Clients shared information about their children or grandchildren, and professionals shared information about their private lives, such as holiday plans or major events such as a wedding. Client 29, receiving mental healthcare: *“She remained professional, but also... well... she did get that little bit closer to you.”* Some clients said that a professional felt like a sister or a friend. Some clients appreciated physical contact with professionals. Clients talked about instances such as a professional touching a client’s cheek, placing an arm around the client’s shoulder, a hug to comfort, and a hand on the clients’ knee as support at a stressful dentist meeting. Professionals also described the added value of physical contact for support. Other clients or care professionals preferred keeping a distance. Client 7, intellectually disabled: *“It’s not right, because that’s simply getting a touch too close.”* One professional described a client who systematically came physically too close, into his comfort zone; the professional therefore adopted a more distant and careful position with this specific client.

### Contextual level

Three generic determinants of a care relationship were mentioned at the contextual level: continuity, lack of time, and limited financial budget and facilities.

- *Continuity*

For continuity at the contextual level, clients and professionals described several aspects. Changes in primary professional, temporary professionals and rotation policies of staff decreased the sense of continuity in care relationships. Professionals explained that a fixed designation of one professional to one client will make it easier for clients to show feelings and preferences. Some clients felt anxious that a professional would quit or would not be assigned to them anymore. Second, cooperation between professionals was related to continuity, which involved good communication and availability of backup for primary professionals. Client 5, intellectually disabled: *“If there’s anything, she writes it down. If X is on holiday, she just writes everything in the app. When X comes back, everything that’s happened is all nicely written down.”*

- *Lack of time and limited financial budget*

Some professionals felt there was a lack of time for paying real personal attention to every client. Limited budgets were also mentioned, for example when a client is temporarily in a hospital or dies, the finance ends immediately while assistance to family members might still be desirable. There were experiences in which there was no budget for uncalculated relaxing activities. Waiting lists were also mentioned by professionals as hindering care relationships.

- *Facilities*

Professionals described facilities as both a promoting and a restricting determinant of care relationships. Facilities that help are training opportunities and home automation devices. Facilities that hinder are old buildings without individual facilities such as a private bedroom, bathroom and television, a poor atmosphere in the building, malfunctioning electronic devices and Wi-Fi, limiting electronic medical record systems (EMR systems), and a lack of training opportunities for professionals.

### Other actor: family of clients

Family was another actor or level that appeared in all focus groups with professionals as influencing the individual relationship between a client and a professional. As family is often part of a client’s social network, often provides informal care for a client, and is the legal representative of a client in some cases: a professional needs to stay on good terms with family members of clients. A professional described family as the third point of a triangle that includes clients and professionals as well. Family members can facilitate a client in a care relationship, but can also have a hindering role. One example given concerned family members who had excessive demands that could not be met, while the client had alternative wishes but did not want to insist. When the interests of clients and their families are in conflict, this can put the professional in a difficult bridging or in-between position. Specifically the professionals who provided care to older people who are mentally or physically frail said that they feel that family members are sometimes ‘inspecting’ them.

### Determinants specific to one client group

Five determinants did not come to the fore in all three client groups, but only in one or two. Determinants on contextual level were only mentioned by professionals.

### Client level

- *Function of the care relationship*

The older clients who were mentally or physically frail described several functions of a care relationship. For example, a professional can serve as a welcome interruption, increase sociable conversations, give comfort in grief after losing a loved person, serve as a link to the world outside the care organization, and help people feel at home in the care organization. On the other hand, some clients preferred to have a task-focused relationship, without small talk or exchanging personal experiences. A professional providing mental healthcare had the feeling that the function required from the care relationship has changed to being more task-focused.

- *Cognitive capabilities*

Some clients with intellectual disabilities mentioned that it is important that they can understand a professional’s expressions and vocabulary. Professionals providing mental healthcare felt that some clients with intellectual disabilities comprehend what a professional tries to communicate less easily. A professional providing mental healthcare: *“There are clients, in particular people with some degree of learning disability, where you have to watch how you formulate things very carefully. With autistic people too: you’ve got to be very careful how you phrase things and you may have to watch your step with humour too.”*

### Between client and professional

- *Humour*

Professionals in mental healthcare and clients with intellectual disabilities or mental health issues believed humour, having fun and laughing relate to a good care relationship. A professional providing mental healthcare: *“[...] We’d laugh a bit together at times to keep it all a bit lighter or to put things into a different perspective.”* However, jokes could also hinder a good care relationship. In one instance, a client’s jokes were not appreciated by the professional. In another example, a professional was laughing the whole time, which led to the client feeling that she was not being taken seriously.

### Contextual level

At the contextual level, support from management, clear communication and accountability, and limitation by laws and regulations were found for one or two client groups only.

- *Support from management*

Professionals in mental healthcare and disability care suggested the support and commitment of their direct manager and the board of management are needed if they are to perform their duties properly. A professional providing mental healthcare: *“If something isn’t going smoothly, if you’re in a conflict situation or can’t resolve issues, you need support from your own manager and you need them to be able to escalate it if necessary.”*

- *Limitations by laws and regulations*

The professionals in mental healthcare and disability care believed that the administrative workload for fulfilling the requirements prescribed by Dutch law is limiting direct contact time. Strictly following the rules made within a care organization was also suggested to be a hindrance.

## Discussion

The aim of this qualitative study was to explore the determinants of the quality of the care relationship between clients and professionals in long-term care, which is one aspect of the quality of care. A determinant was defined in an open and inductive manner, as an aspect related to the quality of a care relationship which might determine a care relationship in a positive or negative manner. The study focused on three client groups receiving long-term care, namely clients with mental health problems, physically or mentally frail older adults, and clients with intellectual disabilities. Respondents receiving both inpatient and outpatient care were included. Based on the qualitative data, determinants were categorized at four levels: client, professional, between client and professional, and context.

There was a large amount of overlap between the three client groups in the determinants of the quality of care relationships. This suggests that the current focus in research on care relationships in long-term care, which tends to be specific to a client group, is not needed. This might have implications for the approach of both quality improvement initiatives and future research focusing on the quality of care relationships. It confirms the result of a recent systematic review of the determinants of the quality of care relationships in long-term care: that a substantial number of determinants apply to multiple client groups in long-term care [24].

Although none of the preceding studies included three distinct client groups when studying the quality of care relationships in long-term care, most results are in line with studies focused on one client group [1, 3, 6, 18, 34–36] [2, 21]. This overlap with earlier findings provides confirmatory evidence for the quality of the study findings. The qualitative and inductive approach of the current study made it possible to confirm previously found determinants and identify unknown determinants at the same time. New determinants found in this study at the client level were ‘help request’ and ‘feedback supply’. At the contextual level, ‘facilities’ was added as a new generic determinant for all three client groups, and ‘support from management’ and ‘limitation by laws and regulations’ came to the fore in two client groups.

Determinants described in this study fit in well with current views on relational aspects of care, specifically within the theoretical framework of person-centred care [37, 38] and its core principle of humaneness. Recognition as a human being and being valued by others is essential to all people, it promotes individual dignity [39]. Determinants such as self-determination of a client, a professional’s focus on an individual client and all determinants at the level between client and professional reflect this core value clearly.

The dependency of clients characterizes care relationships in long-term care. It is therefore useful to reflect further on this aspect. A recent publication describes friction in the care relationship as the moment on which dependency is *experienced* as such by clients [40]. The dependency experienced by clients on care provided by professionals sometimes makes it difficult for clients to speak openly about improvement opportunities [41]. This open conversation has to take place in a very safe way in order to encourage a client to provide truthful feedback, in this case on the quality of a care relationship. Clients differ in the degree of feedback they provide to professionals, as was described at the determinant ‘feedback supply’. A more developed care relationship can make it easier for clients to feel open and have their say, as was described by the determinant ‘relationship building’. Determinants on the professional level might also influence the degree to which a client feels invited to share their ideas (e.g. whether a professional is available, takes time, listens well). In this sense, a care relationship is essentially mutual in the manner it is formed and shaped, as it depends on the unique interactions between a client and a professional and their behaviours.

The determinants of the quality of a care relationship as presented in this study can serve as guidance for care professionals to work towards good quality care relationships and prevent negative consequences of dependency. As care relationships are not fixed, but rather variable and fluctuating, they are inherently individually constructed and need to be personalized between each client and professional. Professionals can use the findings as a starting point for a conversation with an individual client to hear what determinants matter to him/her, in order to fine-tune and improve the quality of their care relationship. Moreover, evaluating the quality of an individual care relationship will help care professionals get a clearer picture of specific areas for improvement. The findings can be used to focus quality improvement initiatives on determinants of the quality of a care relationship. This will help focus on issues that matter from the client’s perspective, as is intended if value-based healthcare is to be achieved [42].

This study was performed by three research teams composed of researchers and co-researchers. The contributions of and cooperation with clients as co-researchers was expected to improve the quality of the findings and to lead to recognition among involved co-researchers [25, 30]. During the study, we observed that co-researchers understood and spoke the language of other clients very well, and were able to ask questions and summarize findings in a way that remained close to the understanding and phrasing of the clients interviewed. Moreover, the clients wanted to participate and were willing to tell their experiences in an interview to co-researchers. In interviews, clients were very open. This corresponds to earlier experiences with participatory research [28]. These aspects are likely to improve the internal validity and therefore the quality of the findings. Co-researchers also felt it was meaningful to be able to contribute to the research and to interview other clients. Their involvement created feelings of being useful and working towards a greater goal. This acknowledged co-researchers, as team members empowered themselves and each other [43].

A strength of this study is the relatively large number of clients interviewed. This resulted in a variety of determinants being found in all three client groups. Another strength is that both client and professional perspectives were included in the study. However, we did not aim at saturation within each care organization in terms of the professional perspective due to the focus on the client perspective, which is a potential limitation of the study. Nevertheless, the results for the professional perspective are in line with the results of our systematic review of this topic and some new determinants were found. This indicates that most fundamental determinants were found in the focus groups.

Regarding client participation, we decided to counteract pseudo-participation by sticking to the tasks and activities that co-researchers were really good at and interested in. This article was written by the researchers without consulting the co-researchers. The article was based on reflections upon the results as discussed in the research teams. The co-researchers focused primarily on the findings of their client group, and the intergroup comparison between the sub-studies was carried out by two researchers. The co-researchers took a training course to learn and practice the basic skills needed for semi-structured interviewing. There were still some moments where a co-researcher asked a closed question that directed a client towards a particular answer, potentially reducing the quality of the interviews. Due to the fact that interviews were conducted by pairs consisting of one co-researcher and one researcher, the researcher was able to reformulate questions or ask for more clarification or examples in these occasions.

One limitation of this study concerns the respondents selected for the interviews. This study focused on clients who were able to speak about their care relationship experiences. This means that some groups in long-term care were not interviewed and excluded from the target group on beforehand, i.e. people with a severe disability or severe forms of dementia. The consequence is limited generalizability of the determinants found to the least articulate client groups who also receive long-term care. Furthermore, the respondents received or provided care within three care organisations. The results from the present study do not attempt to portray a general opinion on satisfaction on care relationships within the care organisations but instead were an exploration of determinants related to the quality of care relationships from a client perspective and a professional perspective. Although organisation cultures may differ, it is likely that the determinants of care relationships are transferable for this study purpose to other care organisations providing long-term care as saturation was achieved in the interviews with clients. Also the variation of client characteristics increased the transferability of the findings, such as type and intensity of care, sex, age, and care setting.

The study findings give four directions for future research. The main findings of this study expand the knowledge of determinants of the quality of a care relationship between client and professional in long-term care. A follow-up study might give a better picture of the use of these determinants for improving the quality of individual care relationships. Furthermore, it is not possible on the basis of this qualitative, inductive study to provide insights into which determinants are most influential on the quality of a care relationship, according to clients and professionals. It is also not yet known what determinants are most and least often met in existing care relationships. Future (quantitative) research might provide more insights into these issues. Lastly, the influence of the families of clients on the client-professional relationship was only described briefly in this study. Future researchers might look more closely at this triangle between client, professional and family in order to examine in what ways a client’s family also influences the quality of care relationships.

## Conclusion

The results of this study show that the majority of determinants of the quality of care relationships are similar for all three client groups of long-term care that were studied: clients with mental health problems, physically or mentally frail older adults, and clients with intellectual disabilities. This finding suggests that a specific focus on a single client group is not needed when studying, monitoring and improving the quality of care relationships. The determinants discovered can be used by care professionals, client councils and other people involved in quality improvement initiatives or when evaluating the quality of an individual care relationship.

## Additional file

Additional file 1: Topic list interview (DOCX 15 kb)

## Data Availability

The data analysed in the current study is available from the corresponding author on reasonable request.
