# Supplementary material for: Determinants of the quality of care relationships in long-term care - a participatory study
Source: BMC Health Serv Res. 2019 Jun 14;19:389. doi: 10.1186/s12913-019-4195-x (PMC6570956; doi:10.1186/s12913-019-4195-x)
Supplement: Supplementary file 1 — Topic list interview (DOCX 15 kb) [file 12913_2019_4195_MOESM1_ESM.docx]

**Additional file 1: Topic list interview**

Part A

1. Which professional from (care organization) do you deal with most?
2. What does this professional (name) help you with?
3. What is the care relationship with this professional (name) like?
4. Why is the relationship (good or not so good)?
5. What else can you say about the relationship?

*[Ask questions 4 and 5 two or three times and keep following them up. You can stop when the client has nothing further to add.]*

- Any more questions? Have a look at the crib sheet.

Part B

*[If the positive points have been described first, we now move on to the less positive aspects.]*

*[If the negative points have been described first, we now move on to the positive aspects.]*

*First positive, now negative:*

You’ve told us a lot about how pleasant your relationship with your professional is.

1. Are there also things about the relationship with your professional that aren’t so nice for you?
2. What other aspects make the relationship less pleasant?

- Any more questions? Have a look at the crib sheet.

*First negative, now positive:*

You’ve told us a lot about what you don’t like about your relationship with your professional.

1. Are there also things about the relationship with your professional that are nice?
2. What other aspects make the relationship pleasant?

- Any more questions? Have a look at the crib sheet.

9. Is there anything else in your relationship with (name of professional) that you’d like to mention?

-------------------------------------------------------------------------------------------------

*Go/no-go point for the researcher to decide if we’re going to ask questions about a second relationship. Rounding off the interview? => Go to “Completion”. Ask about a second professional? => Go to Part C.*

Part C

You’ve told us about the care relationship with (name). We’d also like to hear about your experiences with another professional, where the care relationship is different (*If a good care relationship has been described first, we now move on to a care relationship that is experienced as not so good, and vice versa).*

1. Do you have a professional from (care organization) with whom you have a (good/less good) care relationship?
2. What does this person guide you in?
3. What is your relationship with this professional (name) like?
4. What makes this relationship different from the relationship with the first professional?
5. What else would you like to say about this relationship?

*First positive, now negative:*

You’ve told us a lot about how pleasant your relationship with your professional is.

1. Are there also things about the relationship with your professional that aren’t so nice for you?
2. What other aspects make the relationship less pleasant?

- Any more questions? Have a look at the crib sheet.

*First negative, now positive:*

You’ve told us a lot about what you don’t like about your relationship with your professional.

15. Are there also things about the relationship with your professional that are nice?

16. What other aspects make the relationship pleasant?

- Any more questions? Have a look at the crib sheet.

1. Is there anything else that you’d like to tell us about the relationships?

**Crib sheet – probing deeper**

*…for explanation*

- What do you think is the reason for that?
- What causes that?
- How can you tell?
- Why (or why not)?
- What did you think then?
- How did that make you feel?
- Why do you think that’s needed?
- At what point do you notice that?
- From what moment does that happen?
- Why is that so important for you?
- How did that happen, exactly?
- Can you say a bit more about that?

*…for examples*

- Do you have an example of that?
- Can you give an example of that?

*Positive*

- What makes that so nice?
- Why does that make you feel good?

*Negative*

- What makes that so awkward?
- Why is that so annoying?

*Extra questions – pick a few*

- When did you start to feel a bond with your professional?
- Do you get the feeling that your professional listens to you?
- Do you trust your professional?
- Can you go to your professional if you have problems?
- If there are things that you don’t like, can you say that to your professional?
- Could you describe a moment when you did (or did not) understand your professional properly?
- Do you get the feeling that your professional sticks to what the two of you have agreed upon?
- How do the discussions about the personal plan affect the bond with your professional?
- Does your professional help you to achieve what you want to achieve?
- How does having several professionals at the same time feel for you?
- Do you think it’s important that you have fixed, designated professionals?
- You live (in your own accommodation/at a care organization). Do you think that this affects your relationship with (name of professional)?
